# Supplementary material for: Proteotranscriptomic Profiling of the Toxic Mucus of Kulikovia alborostrata (Pilidiophora, Nemertea)
Source: Toxins (Basel). 2024 Dec 26;17(1):5. doi: 10.3390/toxins17010005 (PMC11768458; doi:10.3390/toxins17010005)
Supplement: Supplementary file 1 [file toxins-17-00005-s001.zip › Supplementary File S4.zip!/ORF 006024 LINALB/230688690e3e92c6/dom_report.pdf]

Phyre2

Email	vas9i-kz@mail.ru
Description	ORF_006024_LINALB
Date	Fri Apr 7 01:53:58 BST 2023
Unique Job ID	230688690e3e92c6

Domain analysis

Rank	Aligned region
1	c6av8A_
2	c7rz3A_
3	c5t4rA_
4	c5we3A_
5	d1qk7a_
6	c6f61A_
7	c7skcA_
8	c1s6xA_
9	c1qfdA_
10	d1clvi_
11	c8feyA_
12	d1en2a2
13	d1ehda2
14	c7namB_
15	c6f8eA_
16	c2n86A_
17	c1zwuA_
18	c2m9lA_
19	c2a2vA_
20	c2jtbA_
21	
22	
23	
24	
25	
26	
27	
28	
29	
30	
31	
32	
33	
34	
35	
36	
37	
38	
39	
40	
41	
42	
43	
44	
45	
46	
47	
48	
49	
50	
51	
52	
53	
54	
55	
56	
57	
58	

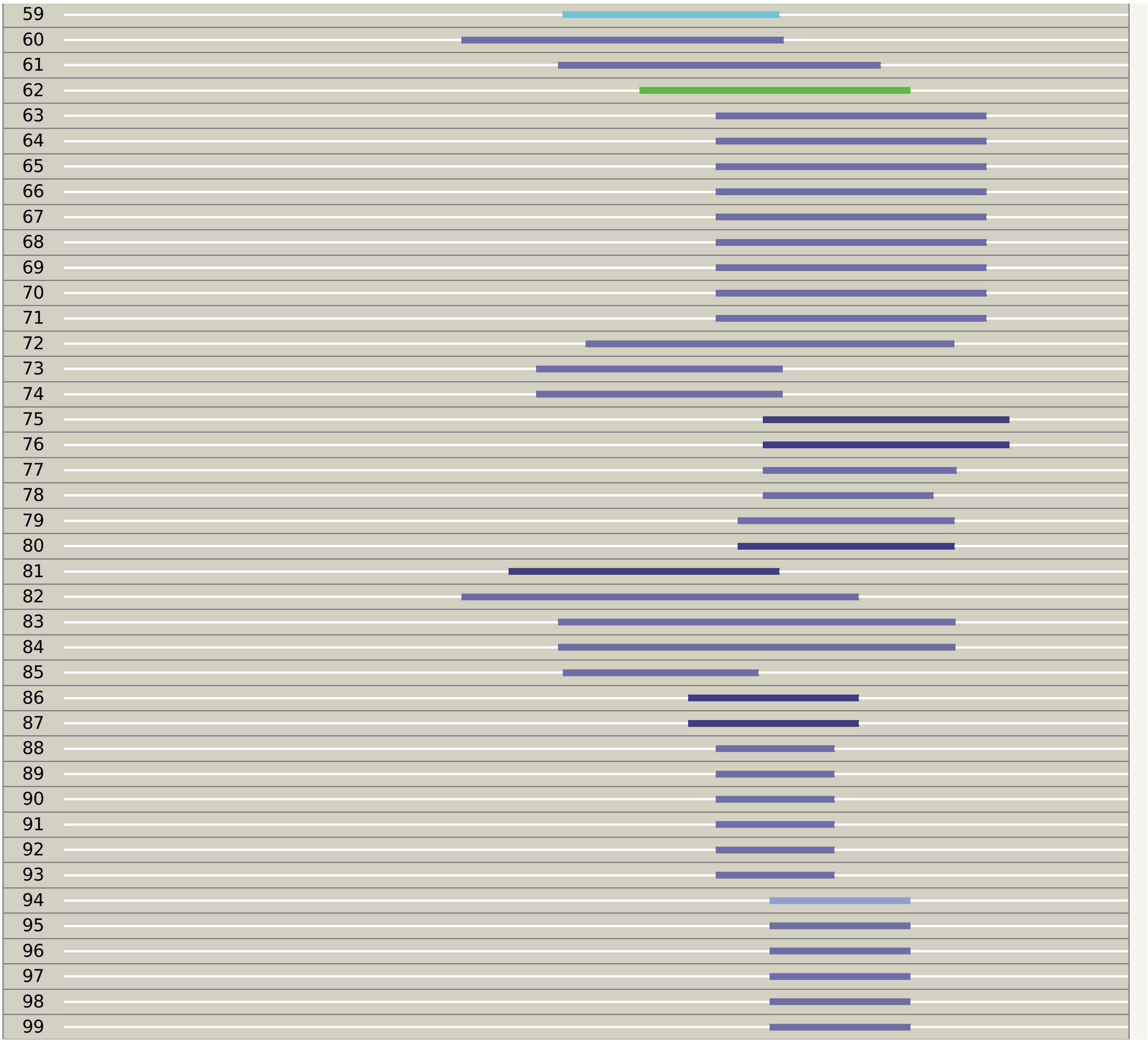
59
60
61
62
63
64
65
66
67
68
69
70
71
72
73
74
75
76
77
78
79
80
81
82
83
84
85
86
87
88
89
90
91
92
93
94
95
96
97
98
99
